# Comparison of Physicochemical Changes and Water Migration of *Acinetobacter johnsonii*, *Shewanella putrefaciens*, and Cocultures From Spoiled Bigeye Tuna (*Thunnus obesus*) During Cold Storage

**DOI:** 10.3389/fmicb.2021.727333

**Published:** 2021-10-29

**Authors:** Xin-Yun Wang, Jing Xie

**Affiliations:** ^1^Shanghai Engineering Research Center of Aquatic Product Processing and Preservation, Shanghai, China; ^2^Shanghai Professional Technology Service Platform on Cold Chain Equipment Performance and Energy Saving Evaluation, Shanghai, China; ^3^National Experimental Teaching Demonstration Center for Food Science and Engineering Shanghai Ocean University, Shanghai, China; ^4^College of Food Science and Technology, Shanghai Ocean University, Shanghai, China

**Keywords:** Bigeye tuna (*Thunnus obesus*), spoilage potential, *Acinetobacter johnsonii*, *Shewanella putrefaciens*, muscle microstructure, bigeye tuna

## Abstract

This study investigates the physicochemical changes and water migration of *Acinetobacter johnsonii* (A), *Shewanella putrefaciens* (S), and cocultured *A. johnsonii* and *S. putrefaciens* (AS) inoculated into bigeye tuna during cold storage. The physicochemical indexes [fluorescence ratio (FR), total volatile base nitrogen (TVB-N), thiobarbituric acid (TBA), trimethylamine (TMA), peroxide value (POV), and pH] of bigeye tuna increased cold storage. A significant decrease in trapped water was found in the AS samples, and direct monitoring of the water dynamics was provided by low-field nuclear magnetic resonance. Samples inoculated with *A. johnsonii* and *S. putrefaciens* also induced the degradation of myofibrillar proteins and weakness of some Z-lines and M-lines. Higher values of physicochemical indexes and water dynamics were shown in the coculture of *S. putrefaciens* and *A. johnsonii* than in the other groups. Therefore, this paper reveals that the coculture of *A. johnsonii* and *S. putrefaciens* resulted in a bigeye tuna that was more easily spoiled when compared to the single culture. This study provides insight into the spoilage potential of *A. johnsonii* and *S. putrefaciens* during cold storage, which further assists in the application of appropriate technologies to keep the freshness of aquatic foods.

## Introduction

Bigeye tuna (*Thunnus obesus*) is one of the most abundant abyssal pelagic fish species throughout the world. As the demand for bigeye tuna increased, bigeye tuna have gained much attention for their high nutritional value (Wang and Xie, 2019; Andrews et al., 2020). It is well known that bigeye tuna is known for its high-quality source of animal protein and low fat. Bigeye tuna spoilage is influenced by high water content and microbial activities (Wang and Xie, 2020a). Through low-field nuclear magnetic resonance (LF-NMR) analyses, the water migration and content of bigeye tuna were non-invasively and accurately monitored during cold storage (Sun et al., 2019; Wang and Xie, 2019). T_2_ transverse relaxation time was correlated with physicochemical parameters, such as water-holding capacity, TCA-soluble peptide, hardness, chewiness, TVB-N and *K*-value (Wang and Xie, 2019). This correlation was developed to estimate the quality of surf clams (Wang et al., 2020a), sea cucumbers (Tan et al., 2018), vegetables, fruit (Sun et al., 2019), and beef (Cheng et al., 2019). All the results indicate that LF-NMR is an effective method to monitor water migration and predict quality changes of food during cold storage. Due to the water loss and storage temperature used, fish quality deterioration is generally related to structural changes in muscle that are also induced by lipid oxidation, protein degradation, and proteolysis (Mousakhani-Ganjeh et al., 2016; Sobieszczańska et al., 2020). The quality deterioration of bigeye tuna results in reduced texture, and nutritional value contributes to harmful components that may even cause health issues for humans. To keep aquatic food fresh, cold storage is a convenient and effective method that can slow microorganism growth and endogenous enzyme activity to prevent aquatic food quality deterioration.

Although cold storage retards the growth of microorganisms, many bacteria can grow and generate spoilage metabolites, which cause pollution of aquatic products called specific spoilage organisms (SSOs) during storage. *Shewanella* spp. and *Acinetobacter* spp. are well-known gram-negative bacteria and have been isolated from aquatic food during cold storage (Odeyemi et al., 2018; Parlapani et al., 2019). These microorganisms were the main contributors of TVB-N, putrefactive flavor, trimethylamine, amines, and proteolysis in aquatic food (Li et al., 2019). Hence, bacterial metabolism plays a key role in evaluating the quality of aquatic food. Many research on aquatic food quality during cold storage has mainly focused on the spoilage potential of *Shewanella* spp. (e.g., Yan and Xie, 2020), *Acinetobacter* spp. (e.g., Carvalheira et al., 2016), respectively. Many bacteria were raised during the early stage of cold storage, while *Shewanella* spp. and *Acinetobacter* spp. tended to become the major SSO during the early stage of cold storage. Previous studies have shown that *S. putrefaciens* has been found to dominate the SSO of aquatic food, such as bigeye tuna (Wang and Xie, 2019) and yellowfin tuna (Susanto et al., 2011) stored at 4°C, which can produce high levels of alcohols, aldehydes, 1-octen-3-ol, esters, alkanes, nitrogen and sulfur, causing strong off-odors. Meanwhile, *S. putrefaciens* can form biofilms that cause the sticky surface of aquatic products and are capable of producing H_2_S (Yan and Xie, 2020). Therefore, the growth of *S. putrefaciens* as an aquatic food spoilage marker is necessary to limit the shelf life at low storage temperatures. In a previous study we demonstrated that *A. johnsonii* was isolated and identified as an SSO from bigeye tuna. We found that the TVB-N, TMA, and pH values to evaluate the spoilage potential of *A. johnsonii* increased as storage time increased (Wang and Xie, 2020b). Moreover, some lipid contents changed dramatically by culturing *A. johnsonii*, which directly accelerated the quality deterioration of bigeye tuna by lipid metabolites (Wang and Xie, 2020a). However, the effect of interactions between the growth and metabolism of two bacteria in aquatic food spoilage has been neglected.

Therefore, this study was conducted to further provide information about the interactions between *A. johnsonii* and *S. putrefaciens* on the quality deterioration of bigeye tuna during cold storage. Bacterial growth, physicochemical indexes, lipid oxidation [thiobarbituric acid (TBA), peroxide value (POV), fluorescence ratio (FR)], protein degradation (proteolytic activities, myofibril protein changes), water dynamics, and muscle structural changes were investigated. Furthermore, the spoilage mechanism of the cocultured *A. johnsonii* and *S. putrefaciens* was elucidated, providing novel insights into the aquatic food spoilage mechanism and inhibiting microbial growth to enhance the quality of aquatic foods for future research.

## Materials and Methods

### Bacterial Strains and Reagents

Bigeye tuna steaks (Zhejiang Fenghui Ocean Fishing Company Ltd., China) were stored at 4°C until spoilage. Twenty-five grams of spoiled bigeye tuna was aseptically collected, mixed with 225 mL of sterile 0.85% NaCl solution and homogenized for 1 min. A 0.1 mL suitable dilution was spread on the surface of plate count agar and incubated at 30°C for 72 h. The two species (*A. johnsonii* and *S. putrefaciens*) were identified by 16S rRNA gene sequences and a VITEK^®^ 2 CompactA system (BIOMÉRIEUX, France) and stored at −80°C. *A. johnsonii* (accession number CP070866) and *S. putrefaciens* (accession number CP070865) were thawed and cultured in tryptose soya broth at 30°C overnight until the maximal concentration (10^8^ CFU/mL) was reached. The coculture was defined as a mixture of equal amounts (v/v) of *S. putrefaciens* and *A. johnsonii*.

### Sterile Fish Juice and Sterile Fish Block Preparation

The preparation of sterile fish juice from the back muscle of bigeye tuna (2 kg) was modified from Jia et al. (2020). Bigeye tuna filets were minced, and distilled water was added to mixed muscle at a ratio of 1:1 (water: fish, w/w). The mixture was boiled for 5 min and filtered after cooling. Then, the filtrate was centrifuged at 4200 r/min for 30 min, and the supernatant was obtained. Each liter of fish juice was supplemented with 1.6 g of trimethylamine oxide, 40 mg of L-cysteine and 40 mg of L-methionine. The supernatant was collected and autoclaved at 121°C for 15 min. A 0.1 mL bacterial suspension was aseptically pipetted into 200 mL of sterile fish juice. The sterile fish juice was stored at 4°C. The fish juice was used for microbiological growth analysis, TVB-N, TMA, pH, TBA, POV, FR, and proteolytic activity analyses.

Sterile fish blocks were prepared from bigeye tuna according to the procedure of Wang and Xie (2020b). Sterile fish blocks were used to determine T_2_ transverse relaxation time, water holding capacity (WHC), and muscle microstructure (scanning electron microscopy and transmission electron microscopy) analyses.

The samples were divided into groups according to the method of Niu et al. (2018); Jia et al. (2020), and Wang and Xie (2020b): (a) A control was prepared by adding sterile water into bigeye tuna blocks/juice supplemented with sterile water, (b) *A. johnsonii* suspensions were independently inoculated into sterile fish blocks/juice with an inoculation level of approximately 5 log (CFU/mL), (c) *S. putrefaciens* suspensions were independently inoculated into sterile fish blocks/juice with an inoculation level of approximately 5 log (CFU/mL), and (d) *A. johnsonii* and *S. putrefaciens* suspensions were mixed at a ratio of 1:1 and then inoculated on bigeye tuna blocks/juice with an initial bacterial level of 5 log (CFU/mL).

### Microbiological Growth Analysis

The TVC of bigeye tuna was measured each day. The TVC was determined according to a previously described method (Wang et al., 2017). The results were expressed as colony-forming units in log CFU/mL.

### Physicochemical Indexes

#### pH, Total Volatile Base Nitrogen, Trimethylamine, Water Holding Capacity Value

The determination of the pH value was performed according to Chen et al. (2019). A tuna sample (5.0 g) was added to 50 mL of distilled water in a stomacher to homogenize at 8000 r/min. Then, the sample was maintained at 4°C for 30 min to obtain a supernatant. The pH of the tuna was evaluated by inserting a pH electrode (Cyberscan Model 510; Eutech Instruments Pvt. Ltd., Singapore).

The determination of the TVB-N value was performed according to Li et al. (2019). TVB-N value expressed as TVB-N mg/100 mL bigeye tuna juice. The determination of the TMA value was performed according to Li et al. (2019). TMA value expressed as mg N/100 mL bigeye tuna juice. TVB-N value and TMA value were measured by an automatic Kjeldahl instrument (UDK159;VELP SCIENTIFICA, Italy).

The WHC value was determined according to Fidalgo et al. (2020) with some modifications. A previous 2 g of bigeye tuna block (*Wa*) was wrapped in two filter papers and centrifuged (3,000 × g, 10 min, 4°C). After centrifugation, the surface water was drained and weighed again (*W*_*b*_). WHC value was calculated using the following formula:


WHC(%)=(1-(Wa-Wb)/Wa)×100%

### Lipid Oxidation of Bigeye Tuna

#### Thiobarbituric Acid, Peroxide Value, Fluorescence Ratio Indexes

The determination of the TBA value was performed according to Fidalgo et al. (2020). The TBA value was measured at 532 nm and expressed as mg malonaldehyde/kg muscle sample. The TBA value was calculated using the following formula:


TBA(mgmalonaldehyde/kg)=A×5327.8

where A_532_ is the absorbance at 532 nm.

The POV value of the bigeye tuna was measured according to the AOCS method (2003). The POV value was expressed as meq oxygen/kg.

The FR of the bigeye tuna assay at 393/463 nm and 327/415 nm was measured according to Nian et al. (2020). FR results were obtained from the following formula:


FR=RF/393/463nmRFnm.327/415

### Protein Degradation

#### Proteolytic Activity

Proteolytic activity was spectroscopically quantified using azocasein as the substrate (Zhu et al., 2015). The samples were centrifuged (12,000 g, 10 min, 4°C) and 100 μL of supernatant was added 500 μL of PBS (50 mmol/L, pH 7.2, Sangon Biotech Co., Ltd., China) and 100 μL of azocasein substrate solution (15 g/L in 50 mmol/L PBS, pH 7.2) to incubate at 37°C for 30 min. The reaction was stopped by an equal volume of 0.5 mol/L trichloroacetic acid standing at room temperature for 30 min. Then, the mixture was centrifuged (12,000 g, 5 min, 4°C) to obtain the supernatant and 1 mol/L NaOH were mixed, and the absorbance at 366 nm with a multiplate reader (Synergy 2, BioTek, Winooski, VT, United States).

### Water Dynamics

The T_2_ transverse relaxation time of the bigeye tuna was determined by LF-NMR and Carr-Purcell-Meiboom-Gill pulse sequences according to the method of Wang and Xie (2019). NMR measurements were carried out with a Meso MR23-060HeI MRI system (Niumag Electric Corporation, Shanghai, China) with a time delay between the 90° and 180° pulses (*τ*-value) of 300 μs. The four groups of bigeye tuna were directly used for T_2_ transverse relaxation times of 0, 3, and 6 days.

### Sodium Dodecyl Sulfate-Polyacrylamide Gel Electrophoresis

Myofibrillar proteins were extracted according to the method of Wang and Xie (2019). The myofibrillar proteins (obtained supernatant) of bigeye tuna were used for Sodium Dodecyl Sulfate-Polyacrylamide Gel Electrophoresis (SDS-PAGE) for 0, 2, 4, and 6 days of storage. Six microliters of each myofibrillar protein sample was loaded in a sample well for SDS-PAGE analysis. The gels were stained with 0.1% (w/v) Coomassie Brilliant Blue R-250 in 50% (v/v) methanol for 0.5 h and destaining solution (acetic acid: methanol: water, 3 V: V:6 V) until the bands appeared to be clear.

### Muscle Microstructure

#### Scanning Electron Microscope

Bigeye tuna samples for SEM were prepared as described previously by Deng et al. (2019). The bigeye tuna samples were prefixed with 2.5% (v/v) glutaraldehyde at 4°C overnight. Longitudinal sections of freeze-dried bigeye tuna samples were prepared for extreme-resolution analytical field emission (Tescan Mira 3 XH, Tescan, Czech Republic).

#### Transmission Electron Microscopy

Bigeye tuna samples for TEM were prepared according to the method described by Bao et al. (2020) with some modifications. Bigeye tuna muscle was cut into small strips (5 mm × 3 mm × 2 mm), and precooled 2.5% glutaraldehyde solid was retained overnight. The samples were fixed with osmium oxide for 2 h and subjected to 30, 50, 70, 80, 90, and 95% ethanol gradient dehydration. Thin sections (70 nm) were stained with uranyl acetate and lead citrate using a JEM-2100 transmission electron microscope (JEOL Co., Ltd, Tokyo, Japan) operating at 200 kV.

### Statistical Analysis

The data for TVC, TVB-N, TMA, pH, WHC, TBA, FR, POV, proteolytic activity and T_2_ transverse relaxation time were plotted using OriginPro 2020b software (Microcal, United States). All analyses were conducted with three replicates. Two-way analysis of variance and Duncan’s test significant difference test using SPSS (IBM Statistical Package for the Social Sciences Ver. 22.0 software, Armonk, NY, United States) and were used to find significant differences at a level of *P* < 0.05.

## Results and Discussion

### Changes in Microbiological Growth

**FIGURE 1:**
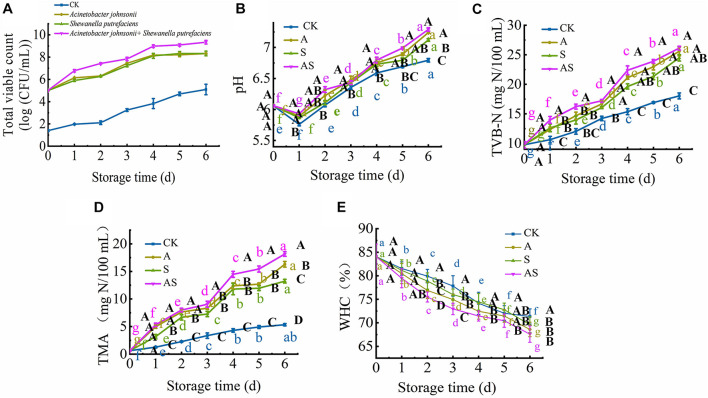
Changes in the total viable count (TVC) **(A)**, pH **(B)**, total volatile base nitrogen (TVB-N) **(C)**, trimethylamine (TMA) **(D)** and water holding capacity (WHC) **(E)** in bigeye tuna with different bacterial groups stored at 4°C for 6 days (each point is the mean value of three determinations). Different capital letters denote significant differences (*P* < 0.05) among bacterial groups at the same storage time. Different lowercase letters denote significant differences (*P* < 0.05) among the different storage times per treatment groups.

Figure 1A shows the microbiological growth of bigeye tuna inoculated with a single SSO and two SSOs throughout the 6-day storage period during cold storage. The initial log TVC values of *A. johnsonii*, *S. putrefaciens* and *A. johnsonii* + *S. putrefaciens* on bigeye tuna were 5.01, 5.22, and 5.02 lg CFU/mL, respectively. The TVC of bigeye tuna showed a significant increase (*P* < 0.05) during cold storage, especially for the group inoculated with *A. johnsonii* + *S. putrefaciens* (from 5.22 to 9.34 lg CFU/mL), while the CK was below 5.0 lg CFU/mL throughout the 6-day storage period and always increased slowly. The *A. johnsonii* group achieved a maximum TVC level and showed a relatively quicker growth rate than the *S. putrefaciens* group during cold storage, suggesting that low temperature was better for *A. johnsonii* to proliferate (Wang and Xie, 2020a). Compared to CK, the TVC of *A. johnsonii* + *S. putrefaciens* group increased significantly, and coculturing was probably one of the key factors accelerating bacterial growth. These results were also similar to previous works that the changes in TVC of the co-culture group significantly increased in refrigerated cooked shrimp (Niu et al., 2018).

### Changes in pH, Total Volatile Base Nitrogen, Trimethylamine, Water Holding Capacity

At the beginning of storage, the pH value of bigeye tuna in the CK, A, S, and AS groups decreased, changing from 6.06 to 5.76, 5.89, 5.86, and 5.93, respectively (Figure 1B). This decreasing trend in pH was observed due to the strong ability of *A. johnsonii* and *S. putrefaciens* to breakdown ATP (Zhou et al., 2019). The pH value of the AS group was significantly higher (*P* < 0.05) than that of the other groups on day 6. This suggested that coculturing *A. johnsonii* and *S. putrefaciens* was more susceptible to bacterial growth at a high pH caused by the production of more alkaline metabolites in coculturing *A. johnsonii* and *S. putrefaciens* (Aghaei et al., 2020; Wang and Xie, 2020a). As the storage time continued to increase, the pH value increased from neutral to the alkaline range, indicating that amines and histamine were produced and autolytic activity occurred during cold storage (Don et al., 2018).

The TVB-N value was utilized to evaluate the freshness of aquatic foods. As shown in Figure 1C, the initial TVB-N value was 9.76 mg/100 mL (day 0) and progressively increased to 18.03 (CK), 25.59 (A group), 24.49 (S group), and 26.12 mg/100 mL (AS group) (*p* < 0.05) after 6 days of storage. Compared to the TVB-N values of the CK, the A, S, and AS groups were all higher than 20 mg/100 mL at day 4, during which the TVB-N values of these three groups reached 21.13, 20.64, and 22.37 mg/100 mL (*P* < 0.05), respectively, which is a higher value than the fresh acceptability for aquatic foods (20 mg/100 mL) (Sharifian et al., 2011). The results showed a sudden increase in the inoculated AS group when bacterial spoilage commenced with the leaching of ammonia.

The spoilage potential and volatile metabolite production among the isolates, muscle protein degradation, reduction of TMAO to TMA and production of off odor (sulfide) due to the presence of proteolytic activity were investigated. The initial TMA value was 0.63 mg/100 mL and increased storage time (Figure 1D). The value of TMA was significantly increased (*P* < 0.05) with an increase in CK, A, S, AS groups from 0.63 to 5.33, 16.29, 13.23, 18.15 mg/100 mL, respectively. Similar results showed that increased cold storage time led to an increase in the TMA value of tuna (Wang and Xie, 2020a). In addition, the results showed that coculturing *A. johnsonii* and *S. putrefaciens* isolated from bigeye tuna resulted in higher TMAO reduction activity. The increase in TMA value might suggest the storage process, which plays in a major role in protein degradation of bigeye tuna causing the strong ability of spoilage potential (Wang and Xie, 2019).

**FIGURE 2:**
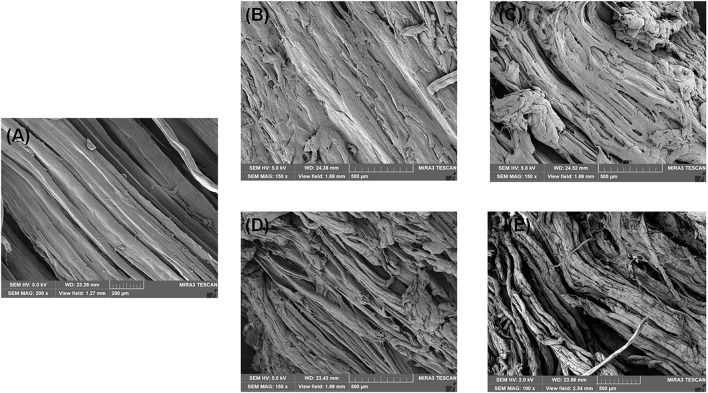
Scanning electron microscopy (SEM) photographs of bigeye tuna muscle stored at 4°C for 0 day **(A)**, stored at 4°C in the control group for 6 days **(B)**, stored at 4°C in the A group for 6 days **(C)**, stored at 4°C in the S group for 6 days **(D)**, and stored at 4°C in the AS group for 6 days **(E)**.

Changes in WHC in four groups are shown in Figure 1E. The initial WHC value was 84.07% and showed a continuous decline with increasing storage time. At day 6, the WHC value significantly decreased to 71.34, 68.60, 69.61, 67.60% in the CK, A, S, and AS groups, respectively. The WHC of the AS group was always lower than those of the other treatment groups. This is probably because spoilage bacteria activities destroyed muscle tissue integrity during cold storage, leading to water loss of muscle (Tan et al., 2018), which was in good agreement with the results of this study (Figure 2).

### Changes in Lipid Oxidation of Bigeye Tuna

#### Changes in Thiobarbituric Acid, Peroxide Value, FR Indexes

**FIGURE 3:**
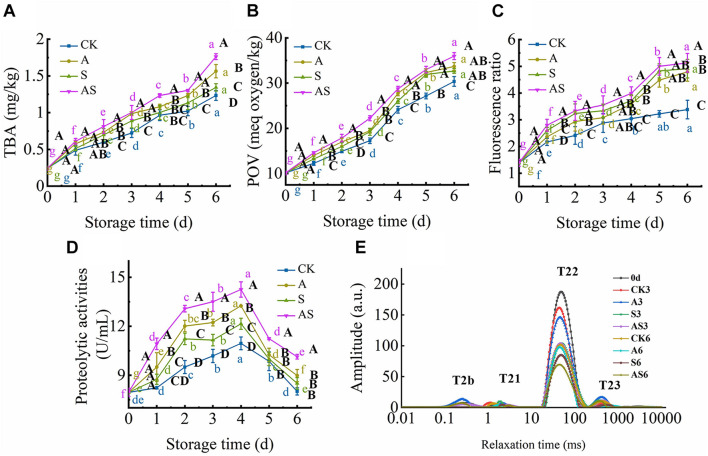
Changes in thiobarbituric acid (TBA) **(A)**, peroxide value (POV) **(B)**, fluorescence ratio (FR) **(C)**, proteolytic activities **(D)** and T_2_ transverse relaxation time **(E)** in bigeye tuna with different bacterial groups stored at 4°C for 6 days (each point is the mean value of three determinations). Different capital letters denote significant differences (*P* < 0.05) among bacterial groups at the same storage time. Different lowercase letters denote significant differences (*P* < 0.05) among the different storage times per treatment groups.

The TBA value is the major index for evaluating the degree of lipid oxidation, reflecting the quality of aquatic foods. The TBA value of the four groups showed a time-dependent increase during the 6 days of storage (Figure 3A). Based on the results of Le et al. (2020), the TBA value was also intensely low due to the very low lipid content in tuna, ranging between 0.24 and 1.76 mg/kg tuna juice over the 6 days of cold storage, although there was a significant increase (*p* < 0.05) at day 0. As the storage time reached day 6, the TBA value of the AS group was increased to 1.76 mg/kg and was higher than that of the other groups, indicating that co-culturing *A. johnsonii* and *S. putrefaciens* produced a large number of aldehydes and ketones by lipid automatic oxidation and hydrolysis, causing the quality deterioration of fish (Chen et al., 2019).

POV, an index of the number of hydroperoxides, evaluates the degree of lipid oxidation at the primary level of aquatic products (Cropotova et al., 2019). Figure 3B shows that the POV dramatically increased with increasing storage time. Moreover, the initial POV value was 10.15 meq/kg and increased to 30.36, 33.67, 32.67, 35.96 meq/kg on the 6th day for the CK, A, S and AS groups, respectively. This result suggested that lipid oxidation products were continuously formed and decomposed into secondary oxidation products, leading to quality spoilage (Wang et al., 2020b). Lipid oxidative degradation of tuna in coculturing *A. johnsonii* and *S. putrefaciens* could accelerate the formation of secondary oxidation products.

Complex formation as a result of the interaction between oxidized lipids and biological amino constituents (mainly proteins, peptides, free amino acids, and phospholipids) was measured by fluorescence, including primary and secondary oxidation products of lipid oxidation. As shown in Figure 3C, there was a continuous increase in the FR value during cold storage. The FR value of the AS group was always higher than that of the other groups. FR was significantly increased after storage for 6 days compared to CK. The results suggested that co-culturing *A. johnsonii* and *S. putrefaciens* expedited lipid oxidation to acquire fluorescent growth due to exposing a variety of aldehydes, which also resulted in bacterial spoilage of tuna.

### Changes in Water Dynamics

T_2_ transverse relaxation has proven to be a precise technique to quantify the changes in water distribution and dynamics of aquatic foods during cold storage and thus helps to explain how these changes are estimated to aquatic food quality using LF-NMR (Wang et al., 2017, 2020a). The T_2_ transverse relaxation is also displayed in Figure 3E. It is noteworthy that four main fractions can be defined as T_2b_, T_21_, T_22_, and T_23_, which were considered strongly bound water (0.1∼1 ms), weakly bound water (1∼10 ms), trapped water (10∼100 ms), and free water (100∼1,000 ms), respectively, which reflected the states of spin-lattice relaxation/spin–spin relaxation inside the bigeye tuna during cold storage (Wang et al., 2020a). The peaks of T_2b_ and T_21_ changed little with storage time in the four groups, indicating that strongly bound water and weakly bound water were strongly related to protein macromolecules, which was in general agreement with Wang and Xie (2019). The distinct decreases in trapped water in the four groups provided a more direct visualization of the water dynamics in bigeye tuna during cold storage. The peak of T_21_ was much higher in CK than in the treated groups, possibly due to the higher WHC in CK to maintain the void volume of the bigeye tuna muscle sample, resulting in water molecules on the muscle that was attached in water during cold storage. Specifically, trapped water showed a significant declining trend in the AS group for 6 days. These results suggested that coculturing *A. johnsonii* and *S. putrefaciens* had an effect on enzyme activity and degradation of the structure of myofibrillar proteins that proteolysis of aquatic food proteins to liberate peptides and enhanced the bioactivity of the protein, causing trapped water within the myofibrillar proteins to be lost. However, the peak of T_23_ increased due to water molecules moving to free water (Figure 3E). This result was proven to rupture the inside myofibrillar lattice membrane, resulting in the mobility of free water being enhanced by bacterial activity.

### Changes in Protein Degradation of Bigeye Tuna

#### Changes in Proteolytic Activity

Proteolytic activity in the bacterium is examined in the presence of exogenous autoinducers, which play a key role in protein degradation and decomposition of protein into small peptides to evaluate the contribution to quality changes (Wang and Xie, 2020c). Proteolytic activity first significantly increased and then decreased (Figure 3D). A similar study showed that cold storage inhibited *A. johnsonii* and *S. putrefaciens* growth to produce protease, while protein was degraded, causing protease activity to increase as storage time increased (Wang and Xie, 2020c). At the end of storage time, small peptides were almost no longer sufficient, causing the proteolytic activity to decrease.

#### Changes in Sodium Dodecyl Sulfate-Polyacrylamide Gel Electrophoresis

**FIGURE 4:**
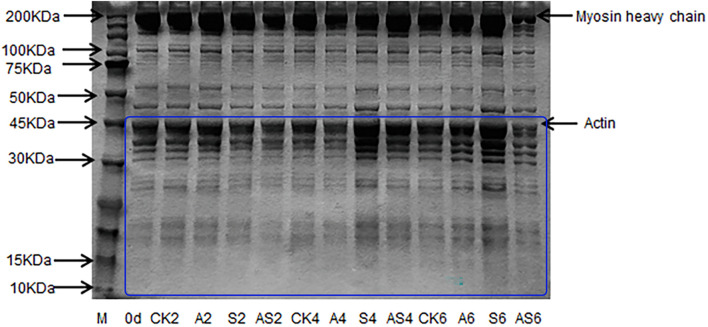
SDS-PAGE of myofibrillar protein of bigeye tuna muscle with different bacterial groups during storage at 4°C. M: molecular weight markers. Lane 0 day: tuna stored for 0 days; lanes CK2, CK4 and CK6: tuna stored for 2, 4, and 6 days at 4°C in the control group; lanes A2, A4, and A6: tuna stored for 2, 4, and 6 days at 4°C in group A, respectively; lanes S2, S4 and S6: tuna stored for 2, 4, and 6 days at 4°C in group S, respectively; lanes AS2, AS4 and AS6: tuna stored for 2, 4, and 6 days at 4°C in group AS, respectively.

Changes in myofibrillar protein in the four groups are shown in Figure 4. In the CK, A, S, AS groups, there was apparent myofibrillar protein breakdown as storage time increased. The intensity of the myosin heavy chain (MHC) of the bigeye tuna was reduced with the concomitant appearance of low molecular-weight fractions in gel following 4 days of storage. A similar result reported that the intensity of MHC in aquatic foods was slightly decreased during cold storage, as described by Wang and Xie (2019). The SDS-PAGE results showed a significant change in the 10–45 kDa AS group; conversely, neither myosin nor actin in the CK group was apparent during storage. These results indicated that low temperature has little effect on protein degradation. One band of approximately 45 kDa became weak in the AS group after preservation for 6 days. Interestingly, the band at 45 kDa in the S group always showed remarkably high intensity. Notable actin muscle content was observed in myofibrillar proteins from the S group. Many degradations of the bands slightly below 50 kDa were found in the AS group, which indicated that myofibrillar proteins gradually faded, including tropomyosin and actin alpha skeletal muscle, due to the microbial activity of coculturing *A. johnsonii* and *S. putrefaciens*, which produced some water-soluble small molecule protein degradation (Cropotova et al., 2019).

### Microstructure Analysis

#### Scanning Electron Microscopy Changes in Muscle Tissues

The SEM images of the bigeye tuna muscle fiber sections are shown in Figure 2. On day 0, well-organized fibers with no damage or rupture of bigeye tuna were observed (Figure 2A). Storage for 6 days for bigeye tuna samples (Figures 2B–E) induced varying degrees of cracks inside the muscle fiber sections. As storage time increased, the muscle myofibril structure was disrupted and disordered due to protein degradation by proteolytic activity and water loss (Bao et al., 2020), which was in accordance with the previous work for Figures 3D,E. For samples stored at 6 days in the AS group (Figure 2E), there was visible deterioration of muscle fibers. These evolutions of myofibril structure might be due to the coculturing of *A. johnsonii* and *S. putrefaciens*, which verified that excess microorganism load could cause the gap between myofibril tissue with large changes in texture or high dehydration of the muscle fibers (Fidalgo et al., 2020). Meanwhile, the muscle fiber changes of the two SSOs in inoculated bigeye tuna samples could also influence the water-holding capacity. Therefore, the SEM results seemingly confirm the results of the T_2_ transverse relaxation time; these disordered arrangements and numerous ruptures of fibers caused water dynamics.

#### Transmission Electron Microscopy Changes Myofibrils Muscle

**FIGURE 5:**
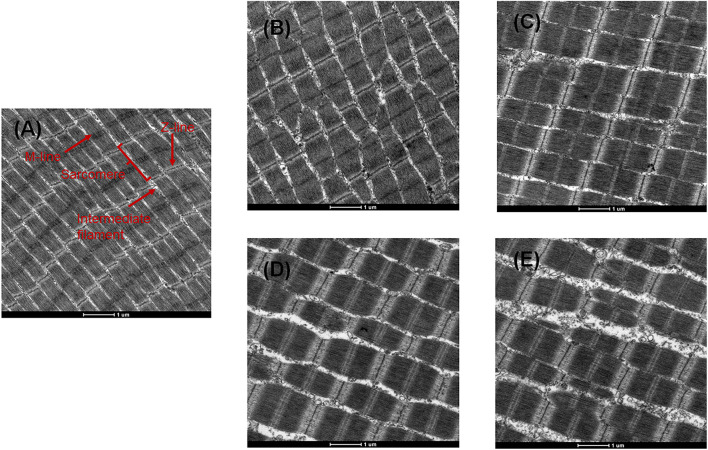
Transmission electron microscopy (TEM) photographs of bigeye tuna muscle stored at 4°C for 0 day **(A)**, stored at 4°C in the control group for 6 days **(B)**, stored at 4°C in the A group for 6 days **(C)**, stored at 4°C in the S group for 6 days **(D)**, and stored at 4°C in the AS group for 6 days **(E)**.

TEM images of myofibril structural changes from bigeye tuna are shown in Figure 5. On day 0, well-organized muscle filaments, cytoskeletons, Z-lines, M-lines, I bands, and A bands were observed. With the extension of storage time, the cytoskeleton showed minor changes between 0 and 6 days in CK, while inter-myofibrillar spaces generally expanded, which was similar to the myofibrilla changes in black carp during cold storage (Bao et al., 2020). After 6 days, A, S, AS samples showed significantly destroyed membranes, and some Z-lines and M-lines were weakened and degraded (Figures 5B–E). Moreover, the increased space to the breakdown of intermyofibrillar links was generally complex and larger in the AS group than in the other groups. For the AS sample stored at 4°C for 6 days, inoculation of myofibrillar proteins with *A. johnsonii* and *S. putrefaciens* caused obvious ultrastructural changes in myofibrils, which were mainly characterized in the thin I band near the Z-lines. The results indicated that *A. johnsonii* and *S. putrefaciens* had synergistic effects to accelerate the breakage of the sarcoplasmic reticulum and severe degradation of protein structures, evidenced by near the Z-line and discontinuous Z-disk.

## Conclusion

The bacterial growth, physicochemical indexes, lipid oxidation, protein degradation, water dynamics and muscle structural changes of sterile bigeye tuna flesh/juice inoculated with *A. johnsonii* (A) and *S. putrefaciens* (S) and coculture with *A. johnsonii*, and *S. putrefaciens* (AS) were investigated. As storage time increased, AS group grew faster than the other groups and had the highest values of TBA, POV, and FR, indicating that coculture with *A. johnsonii* and *S. putrefaciens* had stronger corruption-inducing lipid oxidation. The results showed that the decreases in T_21_ (trapped water) and protein degradation in the AS group during cold storage can be visualized by LF-NMR and SDS-PAGE, respectively. Based on SEM and TEM analysis, microstructural changes in samples inoculated with *A. johnsonii* and *S. putrefaciens* were accompanied by the degradation of myofibrillar proteins (Z-line, M-line, sarcoplasmic reticulum) and the deterioration of muscle fibers during cold storage. These spoilage indexes were observed in lipid oxidation, protein degradation and water loss in bigeye tuna. This study provides an understanding of the spoilage mechanism of coculturing SSOs to help estimate aquatic food quality and shelf-life extension.

## Data Availability Statement

The original contributions presented in the study are included in the article/supplementary material, further inquiries can be directed to the corresponding author/s.

## Author Contributions

X-YW: writing—original draft, data curation, methodology, investigation, and formal analysis. JX: validation, review and editing, project administration, and funding acquisition. Both authors contributed to the article and approved the submitted version.

## Conflict of Interest

The authors declare that the research was conducted in the absence of any commercial or financial relationships that could be construed as a potential conflict of interest.

## Publisher’s Note

All claims expressed in this article are solely those of the authors and do not necessarily represent those of their affiliated organizations, or those of the publisher, the editors and the reviewers. Any product that may be evaluated in this article, or claim that may be made by its manufacturer, is not guaranteed or endorsed by the publisher.
